# New Insights on Cardiorespiratory Fitness in Adults With Systemic Right Ventricles

**DOI:** 10.1016/j.cjcpc.2025.05.004

**Published:** 2025-05-28

**Authors:** Jared Sheridan, Michael Khoury

**Affiliations:** Department of Pediatrics, University of Alberta, Edmonton, Alberta, Canada

**Keywords:** fitness, systemic right ventricles, exercise testing, heart failure

Previous evaluations of functional capacity in adults with systemic right ventricles (sRVs) within a biventricular circulation, largely consisting of those with transposition of the great arteries (TGA) after atrial switch or those with congenitally corrected TGA after physiologic repair, have largely relied on normative data. These studies have consistently demonstrated that cardiorespiratory fitness (CRF) is significantly reduced in patients with sRVs compared with the general population.^1^ Given their relative propensity towards deterioration and progressive heart failure over time compared with those with systemic left ventricles (sLVs), early reductions in CRF may serve as an important prognostic indicator, prompting intensification of surveillance and treatment.^2^ However, the extent to which CRF is reduced in patients with sRVs when compared with adult cardiac patients with sLVs and similar symptomatic burden has not been extensively explored to date.

In this issue of the *CJC Pediatric and Congenital Heart Disease*, Desrosiers-Gagnon et al.^3^ sought to evaluate differences in CRF between adult patients with sRVs within a biventricular circulation and those with sLVs. Through a retrospective single-centre cross-sectional review, adult patients with sRVs and sLVs with a cardiopulmonary exercise test (CPET) performed between 2013 and 2023 were matched at a 1:1 ratio. Exact matching was used for sex and New York Heart Association functional class (class I-IV) based on available records in the medical charts. Coarse matching was used for age (±10 years), body mass index (±5 kg/m^2^), daily dosage of furosemide (≤80 mg or >80 mg), daily dose of spironolactone (≤25 mg or >25 mg), and qualitative matching of systemic ventricular function based on the echo performed at the time of the CPET. As a result of this matching approach, of 256 CPET reports for patients with sRVs and 959 for patients with sLVs, comparisons were undertaken for 24 (21 with TGA and 3 with congenitally corrected TGA) patients with sRVs and 24 with sLVs. For the comparison groups, no differences in CRF were identified between those with sRVs and sLVs, although there were significantly more hospitalizations in the sLV group (58% sLV vs 22% sRV). The authors concluded that patients with sRVs have a reduction in CRF that is comparable to those with sLVs when matched for important demographic and clinical characteristics. Importantly, in an exploratory analysis of patients with a CRF termed “optimal” (peak oxygen consumption [VO_2_] >20 mL/kg/min, which notably is still below average for the general population), peak VO_2_ was lower for the patients with sRVs than for those with sLVs, supporting the notion that there are inherent biophysiologic limitations to cardiac responses to exercise for those with sRVs, including reduced stroke volume exercise responses.^4^

The findings in this study are aligned with the findings in an important prior study by Diller et al.,^5^ which found similar CRF measures in a cohort of young adults with congenital heart disease (aged 33 ± 13 years) compared with a cohort of older adults with noncongenital heart disease (aged 58 ± 15 years). Importantly, in a multivariable Cox analysis, peak VO_2_ predicted hospitalization or death, highlighting the importance of serial assessments of CRF via the CPET in populations at risk for advanced heart failure, such as those with sRVs. The current findings by Desrosiers-Gagnon et al. build on prior work by demonstrating similarly reduced CRF measures between patients with sRVs (an important cohort of adult patients with congenital heart disease) and sLVs, even when matched for age. Moreover, the findings in this study of reduced CRF in adults with sRVs support existing preferences in the contemporary eras at many congenital heart centres towards anatomic repairs (resulting in a sLV circulation) vs “physiologic repairs” where an sRV is maintained for children with congenitally corrected TGA. To this end, recent meta-analysis data have demonstrated improved overall and reintervention-free survival for children with congenitally corrected TGA who undergo anatomic repairs vs those who undergo physiologic repairs.^2^

In this study, Desrosiers-Gagnon et al. elected to undertake a rigorous matching approach that they justify as necessary to appropriately compare CRF levels between patients with sRVs and sLVs with similar demographic characteristics and symptomatology burden. However, the results of this study must be interpreted with a degree of caution, particularly for the risk of selection bias. During the study period, 256 sRV and 959 sLV CPETs were performed. However, the analyzed cohort consists of only 24 for each group, indicating that only approximately 4% of the available patient data were analyzed. Based on the inability to match more patients, it would logically follow that these 2 patient groups do not overlap in demographic and/or clinical characteristics significantly. Specifically, it may be possible that, at a given age, the occurrence of advanced heart failure symptoms and functional impairment in a patient with an sLV may be relatively less common than for those with sRVs. Thus, the patients with sLVs who did meet the matching criteria may represent a subcohort with more advanced disease and may not be representative of the broader population of cardiac patients with sLVs. This in and of itself is an interesting line of questioning raised by the authors with this study and is supported by the difference in hospitalization rates between the groups. In addition, the sLV cohort was relatively heterogeneous, including a variety of pathology. These various disease processes may have different natural histories, including variable hospitalization rates and outcomes independent of CRF. The sRV cohort, in contrast, was more uniform, consisting primarily of patients with TGA after atrial switch. Finally, we note that there are nearly double the rate of implantable cardioverter-defibrillators in the sLV cohort and many more pacemakers in the sRV cohort (37% vs 4%). Arrhythmia burden and pacemaker-induced dysfunction (especially as leads are primarily placed into the RV) are potential sources of both functional limitation and reduced New York Heart Association class. Moreover, limitations in heart rate responses to exercise in pacemaker-dependent patients may have had independent effects on CRF parameters, particularly given known limitations in stroke volume responses to exercise in patients with sRVs.^4^

Overall, this work attempts to tackle an important question about comparative CRF in the sRV population and rigorously aims to isolate for this variable through stringent matching. The authors should be commended for this work, while also highlighting the inherent limitation and possible selection bias of only studying a small portion of the available cohort. Further studies should build on this work through prospective evaluations involving serial CPET assessments linked with long-term outcomes. Moreover, additional retrospective studies that are relatively more limited in their matching approach or use propensity score matching across larger (and potentially multicentre) sample sizes may elicit important new insights. Through their study in this issue of *CJC Pediatric and Congenital Heart Disease*, Desrosiers-Gagnon et al. have made an important contribution in this area, highlighting the need for continued clinical and scholarly efforts to support our growing population of adults with congenital heart disease, including those with sRVs within a biventricular circulation.
